# Purification and Preparation of Graphene-like Nanoplates from Natural Graphite of Canindé, CE, Northeast-Brazil

**DOI:** 10.3390/ma18133162

**Published:** 2025-07-03

**Authors:** Lucilene Santos, Alejandro Ayala, Raul Silva, Thiago Moura, João Farias, Augusto Nobre, Bruno Araújo, Francisco Vasconcelos, Janaína Rocha

**Affiliations:** 1 Department of Geology, Federal University of Ceará, Fortaleza 60455-970, CE, Brazil; 2 Structural Crystallography Laboratory, Department of Physics, Federal University of Ceará, Fortaleza 60455-970, CE, Brazil; ayala@fisica.ufc.br (A.A.); s.araujobruno@fisica.ufc.br (B.A.); 3 Department of Metallurgical and Materials Engineering, Federal University of Ceará, Fortaleza 60455-970, CE, Brazil; raullimasilva@outlook.com (R.S.); willame.vasconcelos@alu.ufc.br (F.V.); 4 Educational Center, Federal Institute of Education, Science and Technology of Ceará, Acaraú 62580-000, CE, Brazil; thiagomoura@fisica.ufc.br; 5 Department of Chemistry, State University of Ceará, Fortaleza 60714-903, CE, Brazil; joaomfarias23@gmail.com; 6 Institute of Geosciences, University of Brasília, Brasília 70910-900, DF, Brazil; augusto.goncalves@unb.br; 7 Materials Research Group, Ceará Center for Technology and Industrial Quality (NUTEC), Fortaleza 60440-552, CE, Brazil; janaina.s@fisica.ufc.br

**Keywords:** microcrystalline graphite, mineral characterization, raman spectroscopy, high-purity graphite, graphene nanoplates

## Abstract

In this study, flotation tests were conducted on a laboratory scale using a sample of microcrystalline graphite ore from the Canindé region, Ceará, Brazil. The objective was to investigate the grinding time, reagent dosage, and purification process for obtaining graphene-based nanomaterials. Natural graphite has a stacked planar structure and exhibits polymorphism with rhombohedral, hexagonal, and turbostratic geometries, characteristics that directly influence its properties and technological applications. The results demonstrated that it was possible to obtain rougher concentrate with a graphite carbon content of 23.4% and a recovery of 86.4%**,** using a grinding time of 7.5 min and reagent dosages of 150 g/t of kerosene and 100 g/t of Flotanol D-25. This flotation process resulted in a graphite concentrate with 76.6% graphite carbon content. To increase the purity of the concentrate and expand its industrial applications, the graphite was purified in an alkaline autoclave using the hydrothermal method. In the next stage, acid leaching was performed, and this chemical treatment destabilized the regular stacking of the graphite layers, promoting the formation of graphene-like nanoplates, including monolayer graphene. Thus, the nanomaterials obtained through the process developed in this study have potential for various innovative applications, such as lithium-ion batteries, electric vehicles, and two-dimensional graphene-based materials.

## 1. Introduction

The microcrystalline graphite ore is located in the Canindé region, Ceará, in northeastern Brazil. Geologically, it is embedded in schists and paragneisses of the Central Domain of the northern subprovince of the Borborema Province [1]. Carbon [2], an abundant element in the biosphere, has been known since ancient times. In its native form, carbon mainly occurs as the diamond and graphite phases. Despite having identical chemical compositions (theoretically pure carbon), these minerals exhibit completely different physical, structural, and crystallochemical properties due to the distinct pressure and temperature conditions under which they crystallize. Graphite is stable at petrologically lower temperature and pressure conditions compared to diamond. Consequently, graphite has a less dense crystalline structure than diamond.

Natural graphite deposits are rarely found in a pure state [3]; they usually contain impurities such as silicates, sulfides, iron oxides, or other substances, making purification necessary for high-performance applications [4]. Graphite is an industrially significant mineral due to its chemical composition and unique structure. Its chemical formula is represented by pure carbon (C), with a crystalline structure composed of layers of carbon atoms arranged in a hexagonal network [5]. These layers are held together by Van der Waals forces, allowing them to slide over each other, which gives graphite properties such as solid lubrication and high thermal and electrical resistance. Beyond its importance as a raw material for various industries, natural graphite is a strategic resource for producing advanced materials. With the growing advancements in nanotechnology and electronics, the demand for high-purity graphite has increased significantly. Obtaining a high-quality graphite concentrate is essential for manufacturing high-performance products, including lithium-ion batteries, supercapacitors, and conductive materials [6].

Various methods are employed for graphite purification, including thermal, chemical, and physical processes [7]. The thermal method involves heating at high temperatures to volatilize impurities but requires high energy consumption. Chemical methods use strong acids and bases to dissolve impurities, which can generate harmful environmental waste. Physical beneficiation includes techniques such as gravity and electrostatic separation, which may have limited efficiency in removing finely dispersed impurities. In this context, flotation emerges as a highly effective method, as it allows the selective separation of graphite from impurities. The graphite has good natural floatability and hydrophobicity. Therefore, most graphite processing plants use the flotation method to purify graphite ore, that is, adding a series of flotation reagents to enrich the graphite ore in the gas–liquid interface to achieve separation from impurity minerals. The graphite flotation method can make the grade of graphite carbon reach 75–90%, or even about 95% [8].

Graphite has widely diversified technological applications due to its structural and electronic properties. Its transformation into nanomaterials, such as graphene, further expands its potential applications in fields such as advanced electronics, biomedical devices, sensors, and energy storage. Graphene is obtained by exfoliating graphite layers [9], resulting in atomic-thin sheets with exceptional properties such as high mechanical strength, excellent electrical and thermal conductivity, and a large surface area. This transformation process can occur through mechanical, chemical, or electrochemical methods, with material stability being a critical factor for its applicability. Graphene nanoplates, which are intermediates between graphite and monolayer graphene, represent a promising alternative due to their ease of production and superior stability in various applications [10].

Among the methods used for graphene production, acid leaching stands out, as it selectively removes impurities and destabilizes graphite layers. This process uses acidic solutions to oxidize and separate individual graphene layers, resulting in materials with high purity and controlled structural properties. Acid leaching offers significant advantages, such as better control over graphene morphology and its properties. Additionally, this technique enables the production of graphene nanoplates with a high degree of crystallinity, enhancing their electrical and mechanical properties, making them ideal for applications in electronic devices and structural composites [10].

The exploration and beneficiation of graphite in the Canindé region, Ceará, have great potential to generate high-value-added products and boost local socioeconomic development. The production of graphene nanoplates from graphite extracted in the region can foster new industries focused on advanced technologies, such as high-capacity batteries, flexible electronic devices, and multifunctional coatings. Furthermore, developing a local production chain based on graphite purification and nanomaterial production could attract investments and create skilled jobs, strengthening the regional economy and promoting sustainable growth [11].

Graphene-like products obtained by exfoliating this graphite have a wide range of applications and can be used in composite materials (incorporating graphene into polymers [12] and other materials significantly improves mechanical properties such as strength and hardness, making it useful for applications requiring lightweight and durable materials [13]) and special coatings [14] (corrosion protection [15], flexible displays and sensors, antistatic and antimicrobial coatings, etc. [16]). Due to the challenges of stabilizing graphene, graphite nanoplatelets have been considered a substitute for graphene in various applications. These nanoplatelets may occur naturally, associated with geological shear zones [4], or be obtained through simple beneficiation processes.

This study aims to develop and optimize methods for purifying and preparing graphene nanoplatelets from natural graphite from Canindé. For this purpose, flotation, leaching, and chemical treatment processes will be investigated to obtain materials with enhanced properties for use in strategic sectors. The results of this study are expected to contribute to advancing knowledge on graphite beneficiation and its applications in nanomaterials, opening new technological and industrial opportunities for the region. Additionally, the economic and environmental feasibility of the employed processes will be evaluated to ensure that the conversion of graphite into graphene nanoplatelets occurs sustainably and efficiently.

## 2. Experimental Section

### 2.1. Experimental Methods

The sample preparation was carried out at Lagetec, Department of Geology, Federal University of Ceará (UFC), while the flotation study was conducted at the Laboratory for the Development of Ceramic Materials, Department of Metallurgical and Materials Engineering, UFC. The graphite purification and nanomaterial formation experiments were performed at the Ceará Center for Technology and Industrial Quality (NUTEC). Chemical analyses by X-ray fluorescence and X-ray diffraction were conducted at the X-ray Laboratory, Department of Physics, UFC. Raman spectroscopy and AFM measurements were performed at the Analytical Center and the Structural Crystallography Laboratory, both within the Department of Physics at UFC. Transmission Electron Microscopy (TEM) analyses were conducted at the National Nanotechnology Laboratory (LNNano/CNPEM).

#### 2.1.1. Flotation Experiment

**Figure 1:**
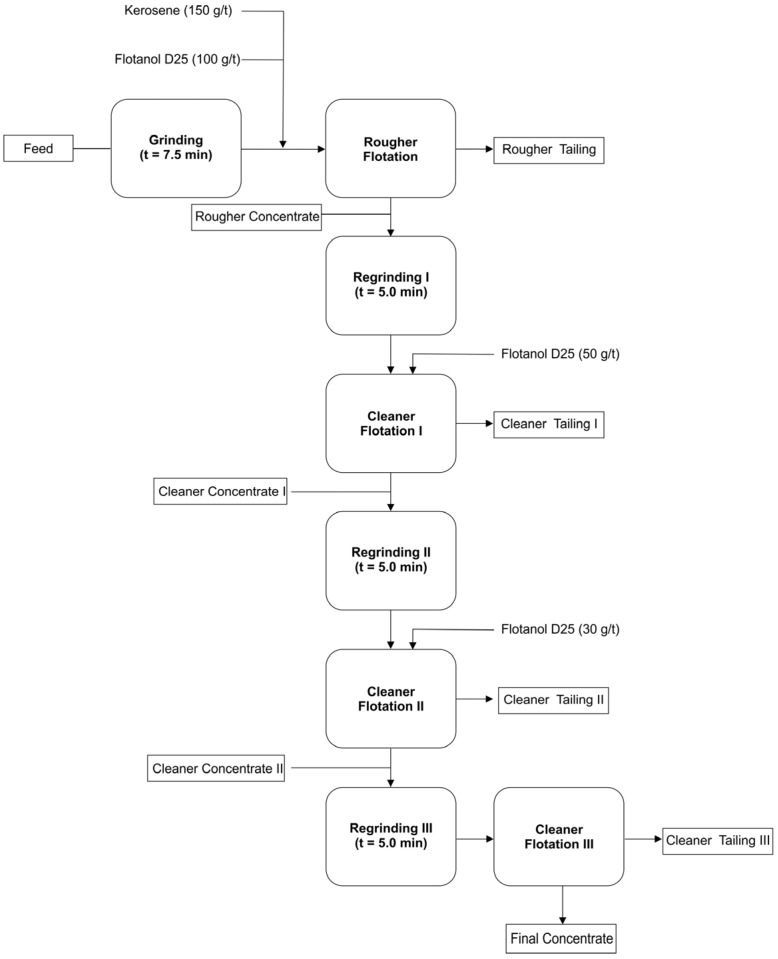
Flowsheet for graphite flotation (final test).

Flotation tests were conducted using a sample of microcrystalline graphite ore. The ore needed to be ground to meet the feed requirements before flotation and was reduced to below 3.35 mm. The focus of the present study was to investigate parameters such as grinding time and reagent dosage to achieve higher graphite recovery through flotation. For natural graphite, a process flow based on staged grinding and flotation is generally used. Laboratory-scale rougher flotation tests were performed to study the grinding time and the dosage of reagents, kerosene (collector) and Flotanol D-25 (frother) [7,8]. Rougher grinding time tests were carried out in a 12″ × 8″ mill using a grinding charge composed of bars with varying diameters. Tests were conducted using different grinding times of 2.5, 5, 7.5, and 10 min while maintaining constant dosages of 200 g/t of collector and 75 g/t of frother. It was observed that the highest graphite recovery was achieved at a grinding time of 7.5 min, as the goal of this stage was to maximize graphite recovery during the rougher flotation. Therefore, this grinding time was adopted for the subsequent reagent dosage tests (Figure 1).

The grinding was performed under wet conditions, with a solids content of 65%. That is, approximately 550 mL of water was added for each 1 kg sample. In this case, the pH considered was the natural pH of water (around 7), since graphite recovery is not significantly affected by pH variations above 4. The decrease in recovery at pH values below 4 is associated with a reduction in the contact angle [17]. The air flow rate in the flotation cell during the tests was approximately 3.0 L/min. The frother Flotanol D-25 was supplied by Clariant (São Paulo, SP, Brazil). The study demonstrated that it was possible to obtain a rougher concentrate with a graphite carbon content of 23.4% and a recovery of 86.4% using a grinding time of 7.5 min and reagent dosages of 150 g/t of kerosene and 100 g/t of Flotanol D-25. Under these conditions, to further concentrate the graphite, a flotation circuit was carried out, consisting of a rougher stage followed by three successive stages of regrinding and cleaner flotation. During the selection process, the target minerals and impurities are gradually dissociated through multiple grinding stages, and the optimal number of grinding and flotation steps is experimentally studied and determined.

#### 2.1.2. Chemical Purification in Alkaline Environment (C-NaOH Treatment)

**Figure 2:**
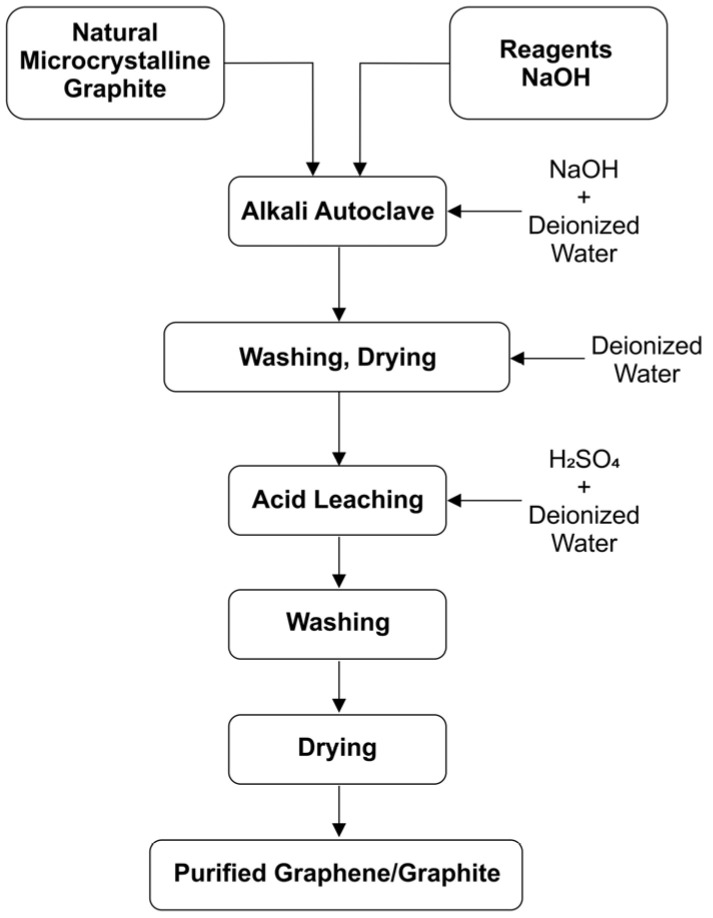
Flowchart of the integrated alkali–acid treatments for graphite purification.

The purification of graphite was carried out through an alkaline treatment [18] in a hydrothermal reactor [19]. Initially, a 1M sodium hydroxide solution was prepared using 61 mL of deionized water. Sodium hydroxide—puriss. p.a., ACS reagent, Ph. Eur. grade, K ≤ 0.02%, ≥98%, pellets (Merk, Darmstadt, Germany). Then, 4.00 g of graphite (C-bulk) was dispersed in the solution, forming a dense and homogeneous suspension. The suspension was transferred to a Teflon vessel, which was properly sealed and placed inside a hydrothermal reactor. The system was then subjected to a thermal treatment in a muffle furnace at 200 °C for 6 h. After this period, the material was cooled to room temperature and subjected to a neutralization process. pH adjustment was performed until reaching pH 7 through successive washings with deionized water. The material was centrifuged and washed to ensure the complete removal of alkaline residues [19]. Finally, the sample was subjected to a drying process in an oven at 70 °C for 24 h, ensuring the removal of residual moisture before subsequent processing and characterization steps (Figure 2).

#### 2.1.3. Leaching in an Acidic Environment (C-NaOH + H_2_SO_4_ Treatment)

The acid leaching of the previously purified material was carried out using sulfuric acid (H_2_SO_4_) in an aqueous medium under magnetic stirring. For this purpose, a 1.0 M H_2_SO_4_ solution was prepared by diluting the required amount of analytical-grade sulfuric acid (98% purity, 18.4 M) in 25 mL of deionized water. Sulfuric acid—for inorganic trace analysis, 93–98%, density: 1.840 g/mL at 25 °C (Merk, Darmstadt, Germany). The powder obtained from the hydrothermal purification step (C-NaOH treatment) was then added to this solution. The mixture was kept under constant magnetic stirring in a heated water bath at 90 °C for 120 min. After the reaction time, the material was centrifuged and washed until reaching pH 7, then dried in an oven at 70 °C until complete moisture removal [18]. The final product was labeled “C-NaOH + H_2_SO_4_ treatment” (Figure 2).

### 2.2. Characterizations

To determine the crystalline structure of the solids, X-ray diffraction (XRD) measurements were performed using a Panalytical^®^ Xpertpro MPD diffractometer equipped (Almelo, The Netherlands) with a Co-Kα radiation tube, operating at 40 kV, 20 mA, and a step size of 2° per minute. The analyzed samples were randomly oriented using the powder method. The 2θ angle range was from 0 to 100°. The interplanar spacing (d) was used to interpret the mineral peaks.

The X-ray fluorescence (XRF) method was used for the chemical analysis of the mineral in powder form, allowing qualitative and semi-quantitative determination of the elements. XRF measurements were collected using a sequential wavelength-dispersive X-ray spectrometer (WDX) RIGAKU ZSX Mini II (Tokyo, Japan), operating at 40 kV and 1.2 mA, with a Pd (palladium) tube capable of performing semi-quantitative analysis of elements ranging from fluorine to uranium.

Fourier-transform infrared spectroscopy (FTIR) measurements were conducted using a SHIMADZU IRXross instrument (Kyoto, Japan), operating in the frequency range of 4000 to 400 cm^−1^, with 64 scans. Absorbance was used as the analytical method, and potassium bromide (KBr) pellets served as the sample support [20].

To identify the physicochemical structure of the analyzed materials [21,22], Raman spectroscopy measurements were performed using an Alpha 300 spectrometer from Witec (Ulm, Germany). A 532 nm excitation laser with a power of 300 microwatts was focused on the sample through a 10× magnification objective lens. Low excitation power was used to prevent sample degradation. The Raman spectra of all three samples exhibited the characteristic peaks of carbon-based materials (D, G, and 2D bands). The Raman spectra were analyzed using the Fityk software (version 1.3.1), where baseline subtraction and spectral deconvolution into Lorentzian components were performed. No significant Raman peak shifts were observed among the different samples. For comparison purposes, all spectra were normalized relative to the G band.

Atomic Force Microscopy (AFM) analyses were also conducted using an Asylum MFP-3D system, operating in intermittent contact mode with silicon tips (NCHR-W, manufactured by NanoWorld, Lonay, Switzerland, characterized by a force constant of 42 N/m and a resonance frequency of 320 kHz). A scanning area of 10 µm × 10 µm was analyzed for each sample. All samples were dispersed in ethanol using magnetic stirring at room temperature for 15 min and subsequently deposited onto a silicon substrate suitable for nanomaterial characterization via Atomic Force Microscopy.

Transmission Electron Microscopy (TEM) analyses were also conducted using a TEM-FEG (JEOL JEM-2100F, Tokyo, Japan) operated at 200 kV. A fraction of the sample (~2 mg) was dispersed using an ultrasonic bath for 2 min and subsequently deposited onto an Ultrathin Carbon Film on Lacey Carbon Support Film, 400 mesh (TEM grid).

## 3. Results and Discussion

### 3.1. Flotation

Graphite recovery was determined based on the mass balance of the process and the graphite carbon content of the concentrates and tailings, calculated by the loss on ignition (LOI) method. First, crucibles were weighed on an analytical balance, and their masses were recorded. Then, using a spatula, a small amount of each sample was added to the respective crucibles (2 g for flotation test concentrates and 3 to 4 g for tailings), and the combined mass of the crucible and wet sample was recorded. After the initial weighing, the crucibles containing the samples were placed in a furnace at 600 °C for 30 min to calcine the volatile matter (organic material). After this step, the crucibles were removed from the furnace and weighed again. The samples were then returned to the furnace for 90 min at 960 °C to burn off the graphitic carbon. Finally, the crucibles containing the dry, carbon-free residue were weighed, and the graphitic carbon content of the samples was calculated.

The microcrystalline graphite ore sample exhibited a graphite carbon content of 6.85%, indicating a significant number of impurities associated with and mixed into the natural graphite. The flotation study results demonstrated that a graphite concentrate with a graphite carbon content of 76.6% and a recovery rate of 64.0% was obtained in the laboratory, increasing the ore purity by removing impurities. However, these results also highlight the need for further studies to improve recovery and achieve a concentrate with a higher graphite carbon content. Given the necessity for more efficient purification, an autoclave purification method was applied in a basic medium, followed by acid leaching.

### 3.2. XRD Analysis

**Figure 3:**
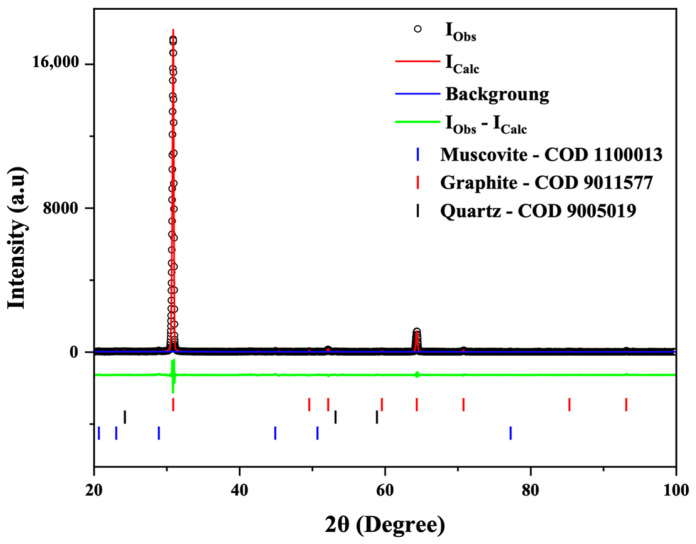
Rietveld refinement of the C-Bulk sample. X-ray diffraction patterns (black circles) together with the result of the calculated pattern obtained with Rietveld refinement (red line). The green line is the difference between the experimental and calculated intensities.

**Figure 4:**
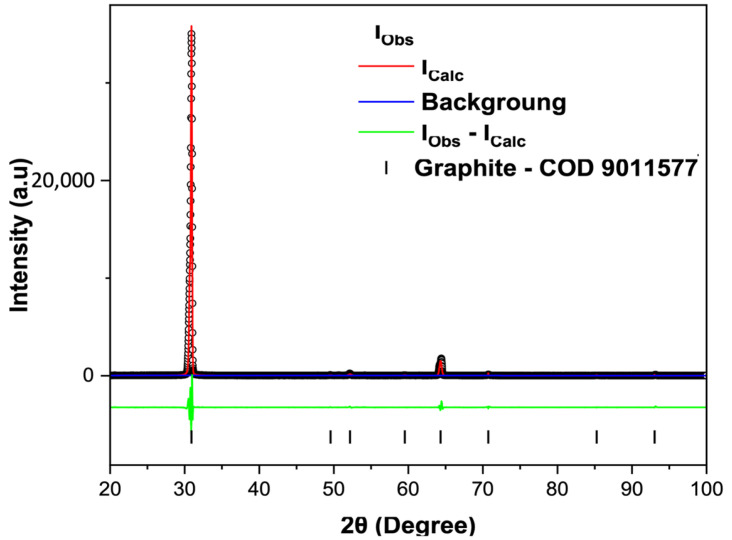
Rietveld refinement of the C-NaOH + H_2_SO_4_ treatment sample. X-ray diffraction patterns (black circles) together with the result of the calculated pattern obtained with Rietveld refinement (red line). The green line is the difference between the experimental and calculated intensities.

For the X-ray diffraction (XRD) analyses, the powders obtained from the following stages were used: C-Bulk (concentrate—after flotation) and C-NaOH + H_2_SO_4_ treatment (final graphene-like obtained). According to the results, graphite is responsible for the highest diffraction peak intensity, with a peak characteristic of graphite (002). Depending on the formation environment, graphite associations may involve muscovite, which was well-matched to most peaks, though with low intensity. The quartz mineral phase is also present. This association of the three minerals is common in deposits, especially when the host rock is a graphitic schist. In all samples, the graphite peak has a 2θ value at 31.5° and an average d_002_ spacing of 3.35 Å [23,24]. Additionally, muscovite and quartz were interpreted in the same peak, coexisting with the graphite peak in the C-Bulk sample and resulting from the pattern calculated by the Rietveld Method using the Crystallographic Information File (Figure 3). Secondary peaks at 1.41 Å, in smaller proportions, were also identified, reinforcing the mineral interpretation for graphite. The XRD refinement pattern for the C-Bulk sample (concentrate—after flotation) consists of graphite with space group P63/mmc (71.4%), quartz with space group P3121 (28.5%), and muscovite with space group C12/c1 (0.1%). The C-NaOH + H_2_SO_4_ treatment sample showed an almost singular graphite peak with space group P63/mmc (100%) and a result of the calculated pattern obtained with Rietveld refinement using the Crystallographic Information File (Figure 4), indicating a well-structured arrangement in the analyzed sample. The lattice parameter and crystallite size from the Rietveld refinements are summarized in Table 1.

The crystalline sizes were estimated using the Scherrer Equation (1), in which β represents the full width at half maximum (FWHM) in radians, adjusted for the instrumental line broadening, while θ denotes the Bragg angle, K is a dimensional shape factor, and λ represents the radiation wavelength.

(1) $$D=\frac{K\lambda }{\beta cos\theta }$$

### 3.3. XRF Analysis

For the chemical analyses of major and minor elements in the whole-rock samples using the X-ray fluorescence (XRF) method, powders obtained from the following samples were used: graphite ore (before flotation), C-Bulk (concentrate—after flotation), C-NaOH treatment (treatment in a basic medium), and C-NaOH + H_2_SO_4_ treatment (graphene-like material).

The chemical composition of major and minor elements in the whole rock of the graphite ore sample shows SiO_2_ = 42.43%, Al_2_O_3_ = 18.88%, CaO = 2.19%, MgO = 1.06%, K_2_O = 7.55%, TiO_2_ = 2.69%, MnO = 2.69%, Fe_2_O_3_ = 23.81%, SO_3_ = 0.34%, and Cl = 0.62%. The C-Bulk sample contains SiO_2_ = 31.02%, Al_2_O_3_ = 15.65%, CaO = 2.44%, K_2_O = 6.32%, TiO_2_ = 2.90%, MnO = 0.54%, Fe_2_O_3_ = 36.16%, SO_3_ = 0.37%, Cl = 0.75%, P_2_O_5_ = 3.67%, and Co_2_O_3_ = 0.18%. The C-NaOH treatment sample presents SiO_2_ = 29.75%, Al_2_O_3_ = 12.88%, CaO = 2.61%, MgO = 1.06%, K_2_O = 5.13%, TiO_2_ = 3.52%, MnO = 0.34%, Fe_2_O_3_ = 39.78%, P_2_O_5_ = 3.73%, and Rh_2_O_3_ = 1.22%. The C-NaOH + H_2_SO_4_ treatment sample exhibits SiO_2_ = 75.65%, Al_2_O_3_ = 3.63%, CaO = 4.70%, K_2_O = 3.71%, Fe_2_O_3_ = 0.86%, and P_2_O_5_ = 11.45%. These values are presented in Table 2.

The chemical composition of major and minor elements in the whole rock of the sample before flotation (raw graphite) corresponds to the mineralogy of microcrystalline graphite ore, which, in addition to graphite, also comprises muscovite and quartz. The high Fe_2_O_3_ content (23.81%) suggests a composition like ferrimuscovite, a variety of muscovite that can occur in high-pressure metamorphic rocks or hydrothermal deposits. The values of SiO_2_ (31.02%), Al_2_O_3_ (15.65%), K_2_O (6.32%), and MnO (0.54%) in the sample after flotation (C-Bulk) decreased, while the MgO content dropped to 0.00%. In contrast, CaO increased to 2.44%, TiO_2_ to 2.90%, Fe_2_O_3_ to 36.16%, and Cl to 0.75%. After flotation, the composition also showed the presence of SO_3_ (0.37%), P_2_O_5_ (3.67%), and Co_2_O_3_ (0.18%). These chemical analysis results for C-Bulk indicate that the flotation of microcrystalline graphite ore is essential for graphite concentration but still requires a more efficient purification process to improve the graphite carbon content.

The chemical composition of the C-NaOH treatment sample (basic medium treatment) compared to C-Bulk shows slightly lower values for SiO_2_ (29.75%), Al_2_O_3_ (12.88%), K_2_O (5.13%), and MnO (0.34%). However, the MgO content increased to 1.06%, while CaO (2.61%), TiO_2_ (3.52%), Fe_2_O_3_ (39.78%), and P_2_O_5_ (3.73%) increased, with the appearance of Rh_2_O_3_ (1.22%) in the composition.

The C-NaOH + H_2_SO_4_ treatment sample presents the highest purity composition among all processing stages, showing a significant reduction in Al_2_O_3_ (3.63%), K_2_O (3.71%), and Fe_2_O_3_ (0.86%), with the complete removal of MgO, TiO_2_, and MnO. Additionally, CaO increased to 4.70%, P_2_O_5_ to 11.45%, and SiO_2_ rose significantly to 75.43%.

The chemical composition of the C-NaOH + H_2_SO_4_ treatment sample demonstrates a strong reduction in impurities such as ferrimuscovite through the reduction and elimination of elements that compose this mica. Since muscovite is the main impurity in microcrystalline graphite ore, the chemical data obtained by XRF and presented in this study indicate a purer natural microcrystalline graphite concentrate.

### 3.4. FTIR Analysis

**Figure 5:**
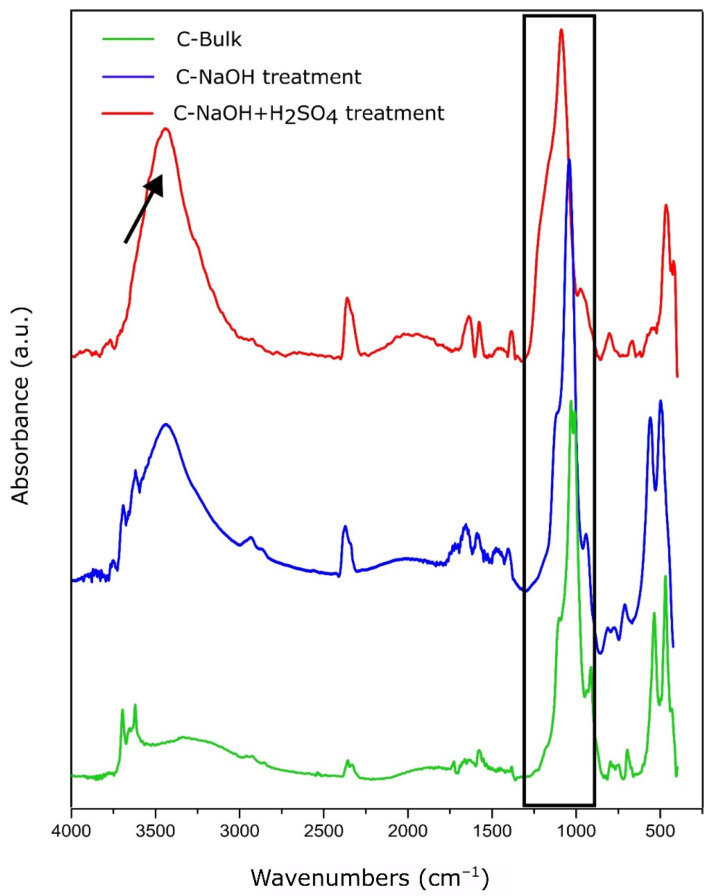
FTIR spectrum of the C-Bulk, C-NaOH treatment, and C-NaOH + H_2_SO_4_ treatment samples. The arrow indicates the highest hydroxyl group density after each step. The region from 900 to 1200 cm^−1^, highlighted in the black box, suggests residual impurities.

Fourier-transform infrared spectroscopy (FTIR) was used to investigate the presence of functional groups throughout the purification and delamination stages of natural graphite. In the raw sample (C-Bulk), a low-intensity spectrum was observed, with prominent bands in the 1000–1100 cm^−1^ region, associated with Si–O–Si and Al–O–Si bonds from phyllosilicates such as muscovite, commonly found in natural mineral-derived graphite. The absence of significant bands in the O–H stretching (~3400 cm^−1^) and C=O (~1720 cm^−1^) regions suggests that the carbonaceous structure is free from oxygen-containing functional groups, supporting the bulk nature of the starting graphite (Figure 5).

After treatment in a basic medium (C-NaOH), an intensified band appears at ~3430 cm^−1^, characteristic of the stretching of free hydroxyl (–OH) groups, which may result from surface hydration. Additionally, a moderate band is observed at ~1620 cm^−1^, attributed to the bending vibration of adsorbed water or to the C=C stretching of the sp^2^ aromatic framework, indicating structural reorganization and possible layer opening [25,26].

The samples treated with H_2_SO_4_ exhibited a pronounced increase in the intensities of these bands, especially at ~3430 cm^−1^, indicating a higher density of hydroxyl groups and enhanced surface water adsorption; at ~1720 cm^−1^, associated with the C=O stretching vibration of carboxylic groups (–COOH), commonly found at the edges of nanoplatelets after mild functionalization; and at ~1030 cm^−1^, which remained present, suggesting residual C–O functionalities, possibly originating from esters or sulfate remnants.

The simultaneous presence of bands attributed to C=O, C–OH, and C=C indicates that the material did not undergo severe oxidation, with the observed spectral behavior being consistent with that of mildly functionalized graphene, especially in few-layer samples where the surface remains partially passivated and the sp^2^ structure is preserved.

### 3.5. Raman Analysis

**Figure 6:**
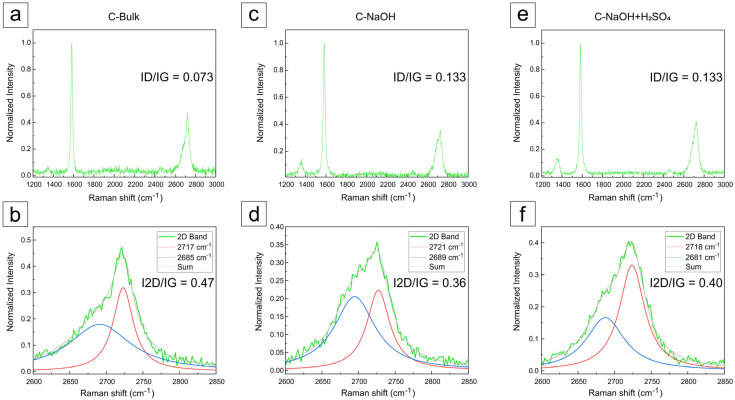
Raman spectra with the deconvolution of 2D peaks (two spectral components). Raman spectra: (**a**) C-Bulk, (**c**) C-NaOH, and (**e**) C-NaOH + H_2_SO_4_. Deconvolution of 2D peaks: (**b**) C-Bulk, (**d**) C-NaOH, and (**f**) C-NaOH + H_2_SO_4_.

The Raman spectra were normalized for all samples. The intensity ratios between the D and G bands are indicated in the graphs, and the main features in the Raman spectra of graphite and graphene are represented by the D, G, and 2D bands. The main bands observed in the Raman spectra of graphitic materials include the G band, associated with the E_2_g vibrational mode of the hexagonal graphite lattice, indicative of ordered sp^2^ carbon; the D band, attributed to structural defects and disorder in the lattice, including edges, vacancies, and functional groups; and the 2D (or G’) band, which is a second-order overtone of the D band and is essential for distinguishing single-layer graphene from few-layer graphene and graphite, based on its shape and relative intensity with respect to the G band (Figure 6).

A typical Raman spectrum of graphene-like materials is primarily characterized by G, D, and 2D bands. The G band arises from the E_2g_ phonon of the hexagonal lattice and is mainly related to sp^2^ coordination. The D band originates from a double resonance process involving lattice defects, such as vacancies or structural disorders. The last 2D band results from a higher-order process involving two phonon scattering processes. Notably, the intensity ratio of the 2D to G band (I2D/IG) can be understood as a fingerprint for distinguishing between monolayer graphene, multilayer graphene, and graphite [21,27]. In contrast, the intensity ratio of the D to G band (ID/IG) offers valuable insights into the atomic structure, including the degree of oxidation and presence of structural defects [28,29]. The corresponding normalized Raman spectra of all samples and the D/G and 2D/G bands are indicated in Figure 6a–f.

**Figure 7:**
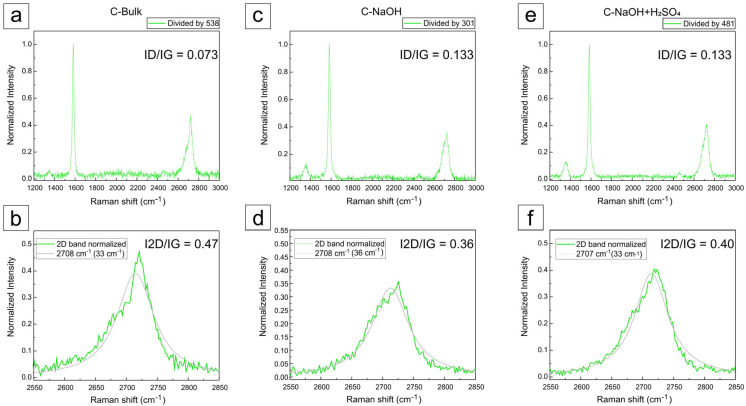
Raman spectra with the deconvolution of 2D peaks (a spectral component). Raman spectra: (**a**) C-Bulk, (**c**) C-NaOH, and (**e**) C-NaOH + H_2_SO_4_. Deconvolution of 2D peaks: (**b**) C-Bulk, (**d**) C-NaOH, and (**f**) C-NaOH + H_2_SO_4_.

For the analysis of the Raman spectra of the produced samples, graphical deconvolution was performed using Lorentzian convolution components using the Fityk 1.3.1 software, after manual baseline subtraction, as shown in Figure 6 and Figure 7.

In the graphite concentrate (C-Bulk), as illustrated in Figure 6a, the G band associated with graphite appears at approximately 1579 cm^−1^, while the 2D band is observed around 2720 cm^−1^, exhibiting a characteristic shoulder typical of graphite [21,28,30,31,32,33,34,35]. After the deconvolution of the 2D band (Figure 6b), two components were revealed at 2685 cm^−1^ and 2717 cm^−1^, which are characteristic of multilayer graphite [32,33,34,35]. The ID/IG ratio of the sample (C-bulk) was 0.073, reflecting a low density of apparent defects, although this was partially masked by the mineral impurities present in the matrix [36].

The purification process in a basic medium revealed significant alteration in the carbonaceous structure throughout the treatment. The sample treated in basic medium (Figure 6c) exhibited similar bands at 2689 cm^−1^ and 2721 cm^−1^ (Figure 6d), still displaying a broad and asymmetric profile, which suggests a partially exfoliated structure. An increase in the ID/IG ratio to 0.133 was observed, indicating the introduction of structural defects, possibly related to partial delamination and functionalization induced by sodium hydroxide at high temperature, as reported in the literature [37,38].

In the final sample, subjected to acid treatment, an intensified and more defined D band at ~1336 cm^−1^ was observed compared to the previous samples (Figure 6e), indicating a higher density of defects or edges in the material. Additionally, bands at 2681 cm^−1^ and 2718 cm^−1^ were observed (Figure 6f), with a very subtle shoulder detected only after spectral deconvolution. The profile is characteristic of stacked structures, supporting the hypothesis of graphene nanoplatelet formation [36,37].

After the acid purification step, the ID/IG ratio remained at 0.133, suggesting that no additional defects were introduced, but rather a reorganization of the layered structure occurred. These findings indicate the formation of few-layer graphene nanoplatelets as a direct result of the acid purification process [39,40].

According to these measurements, the distinction between graphene nanoplatelets and graphene oxide (GO) can be assessed by analyzing the ID/IG ratio, which typically ranges from 0.8 to 1.2 in GO due to its high density of defects and disruptive oxygen-containing groups in the sp^2^ structure. In contrast, the sample produced after acid purification exhibited an ID/IG ratio of only 0.133, indicating few structural defects. Moreover, the 2D band profile was more asymmetric and consistent with few-layer graphene. In GO, this band tends to be significantly broadened or even absent due to the breakdown of the conjugated bonding network [37,38].

The absence of excessive broadening in the G and 2D bands, as well as the I_2_D/IG ratio below 1.5, rules out the presence of pure single-layer graphene. However, the shape of the 2D band (Figure 6) resembles that of graphene with three to five layers [21,27]; this confirms the formation of few-layer, non-oxidized nanoplatelets—i.e., graphene nanoplatelets (after C-NaOH + H_2_SO_4_ treatment). Moreover, the absence of significant broadening in the 2D band indicates that the material did not undergo severe oxidation, which would typically be evidenced by suppression of the 2D band and enhancement of the D band, as commonly observed in graphene oxides [36]. The I_2_D/IG values obtained for the three samples indicate that the analyzed material consists of multilayer graphite nanoplatelets (C-bulk) and few-layer graphene nanoplatelets (after C-NaOH + H_2_SO_4_ treatment), both with more than one layer. These differ from monolayer graphene (I_2_D/IG > 2) and graphene oxide, which typically exhibits a significantly suppressed 2D band due to the disruption of the π-conjugated network. The variation in I_2_D/IG values following chemical treatments reflects the structural and functional changes induced, consistent with those expected from graphene functionalization and reduction processes [39,40].

The acid treatment resulted in an I_2_D/IG value of 0.40, slightly higher than that of the base-treated sample (I_2_D/IG = 0.36). This suggests that the acid treatment may have promoted a mild reduction in previously introduced oxygen-containing groups, partially restoring the conjugated structure of graphene and consequently increasing the relative intensity of the 2D band. However, the value still indicates the presence of a few layers and possible residual defects [36].

The I_2_D/IG ratio of the samples indicates that they are graphene/graphite nanoplatelets with multiple layers and is not the most reliable parameter for determining the exact number of layers. However, the shape of the 2D bands allows for the differentiation of graphene with fewer than five layers [21]. To elucidate the dimensional characteristics of the analyzed samples, additional characterization techniques such as AFM and TEM were required.

### 3.6. Atomic Force Microscopy (AFM) Analysis

**Figure 8:**
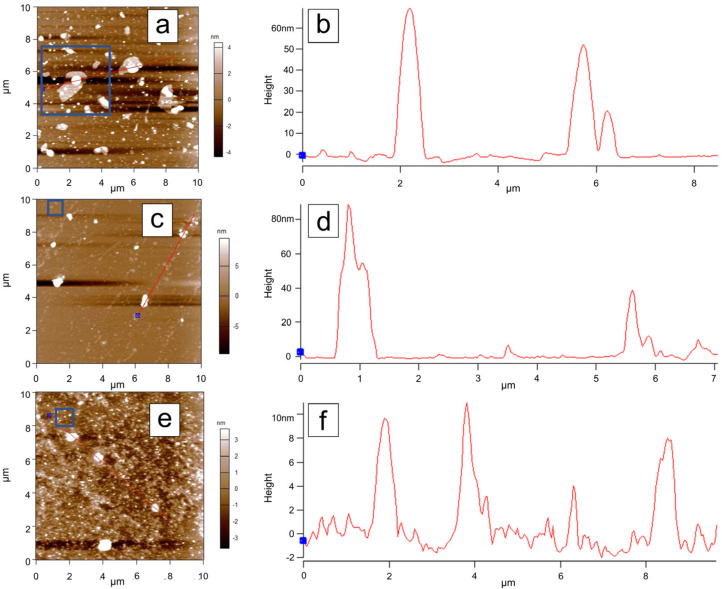
AFM topographic images of dispersions from the three samples. A gradual reduction in the maximum particle size within the scanned regions can be observed. Panels (**a**), (**c**), and (**e**) show the AFM scans of the C-Bulk, C-NaOH, and C-NaOH + H_2_SO_4_ samples, respectively. Panels (**b**), (**d**), and (**f**) present the cross-sectional profiles highlighted in the corresponding AFM scans (**a**), (**c**), and (**e**), respectively. The blue box corresponds to the region identified in Figure 9. The blue dots mark the starting point of the measurement presented in the cross-sectional profiles highlighted by the red line.

To investigate the topographical features of the samples, Atomic Force Microscopy (AFM) measurements were performed on samples dispersed by magnetic stirring in water and deposited onto a silicon substrate. Initially, 10 µm × 10 µm areas were scanned for each sample. The C-Bulk sample (Figure 8a) is characterized by the presence of numerous particles with sizes on the order of tens of nanometers, as well as lamellar structures (Figure 8b), which are indicative of the early stages of nanoparticle formation.

In the AFM scan of the C-NaOH sample (Figure 8c), structures with sizes on the order of several tens of nanometers were also identified, although in smaller quantities compared to the C-Bulk sample (Figure 8d). The large lamellar structures observed in the C-Bulk sample appear to have been fragmented during this purification step, resulting in even smaller structures.

The AFM scan of the C-NaOH + H_2_SO_4_ sample (Figure 8e) indicates greater uniformity in particle size, with the largest structures measuring approximately 10 nm (Figure 8f), representing a significant reduction compared to the previous samples. To better highlight the topographical features of the smaller structures, additional scans were performed in the areas outlined by blue rectangles in each measurement.

**Figure 9:**
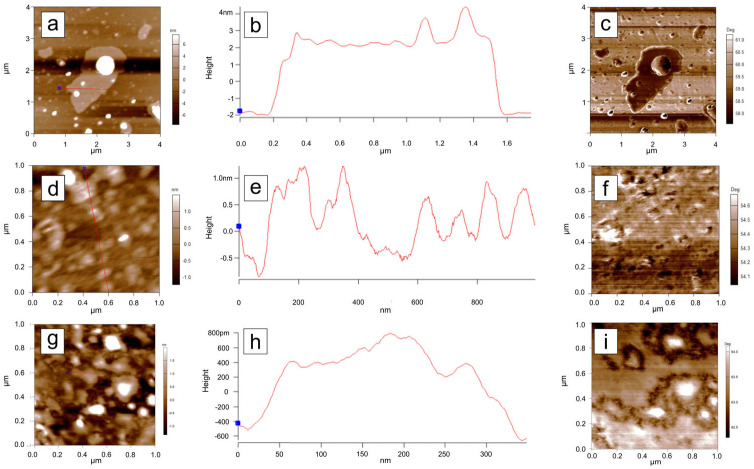
(**a**), (**d**), and (**g**) show the AFM scans of the highlighted in Figure 8. C-Bulk, C-NaOH, and C-NaOH + H_2_SO_4_ samples, respectively. The cross-sectional profiles (**b**), (**e**) and (**h**) extracted from each scan are shown adjacent to the corresponding topographic images, along with the respective phase images, where increased contrast (**c**,**f**,**i**) can be observed in the analyzed regions. The blue dots mark the starting point of the measurement presented in the cross-sectional profiles highlighted by the red line.

For each sample, a high-resolution scan was performed in a reduced area. In the C-Bulk sample (Figure 9a), a large lamellar structure is observed, with a thickness of approximately 4.0 nm (Figure 9b,c), consistent with a graphene-like structure that may have begun to form at this stage, although still in small quantity and within a heterogeneous sample. In the C-NaOH sample (Figure 9d), irregularly sized structures are observed, with heights on the order of 1.5 nm (Figure 9e,f). In the C-NaOH + H_2_SO_4_ sample, structures are observed with heights (Figure 9g) around 1.2 nm (Figure 9h,i).

The phase images corresponding to each topographic image in Figure 9 are shown alongside their respective profile sections in Figure 9c,f,i. Since contrast variations in AFM phase images of graphene samples indicate the presence of different numbers of stacked layers [41], it can be concluded that C-NaOH + H_2_SO_4_ sample is characterized by the stacking of multiple graphene layers, which vary heterogeneously across the analyzed area.

AFM analyses reveal that the purification process resulted in a low-dimensional, heterogeneous sample, characteristic of graphene nanoplatelets, in agreement with the other structural characterizations [36,40].

### 3.7. Transmission Electron Microscopy (TEM) Analysis

**Figure 10:**
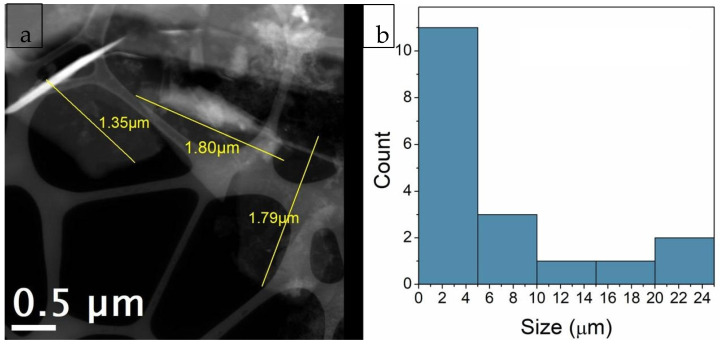
C-NaOH + H_2_SO_4_ sample (graphene and micrographite nanoplatelets): (**a**) HAADF image illustrating the measurement of thin sheet dimensions. (**b**) Size distribution histogram of the thin sheets. This histogram includes only the regions where sheet edges were clearly visible for measurement. Due to folding, tearing, and/or overlapping of the sheets, these measurements represent only an estimate.

**Figure 11:**
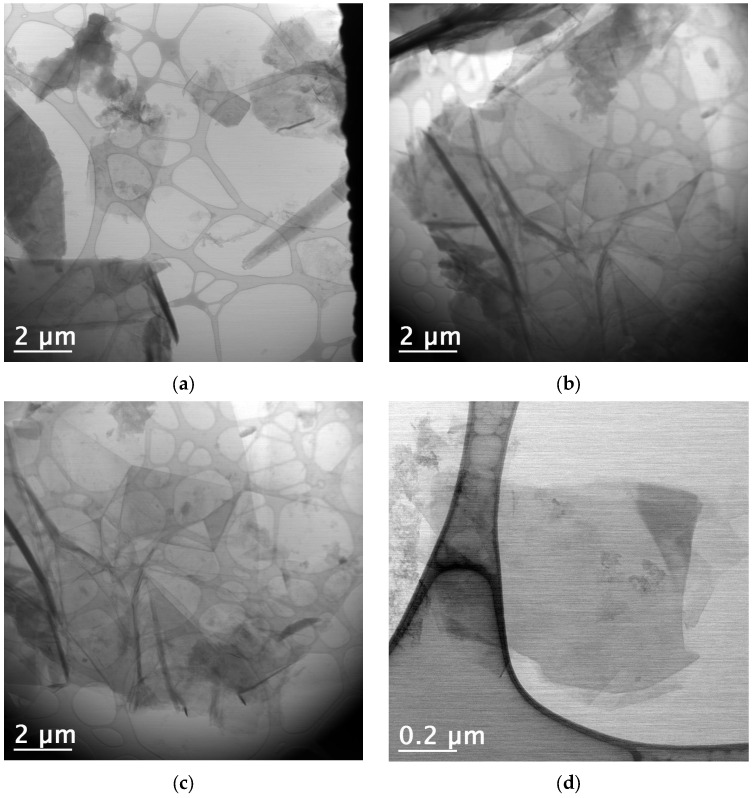
C-NaOH + H_2_SO_4_ sample (graphene and micrographite nanoplatelets)—TEM images of (**a**–**d**): The graphene nanoplatelets (<5 nm) are nearly transparent, suggesting few-layer graphene. The graphene nanoplatelets (>5 nm) and micrographite are less transparent due to the higher thickness.

**Figure 12:**
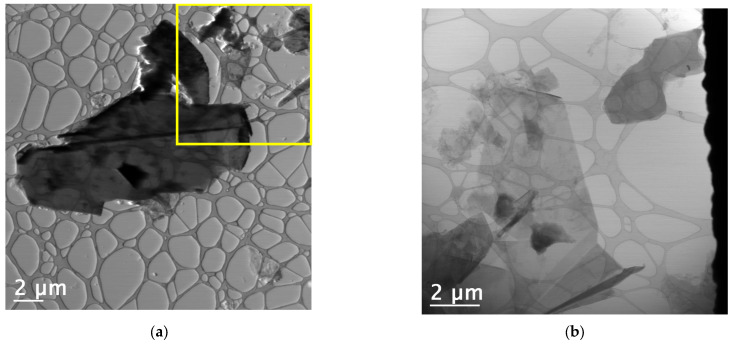
(**a**) BF image of a region of the grid containing the sample, where structures of different sizes can be identified. A large, thin sheet is visible at the center of the image. The yellow rectangle highlights the region with few-layer graphene identified. (**b**) Folded graphene nanoplatelets.

The use of scanning mode measurements (STEM—Scanning Transmission Electron Microscopy) allowed for the measurement of thin sheet dimensions and the determination of their size distribution (Figure 10). All regions were analyzed using both BF (Bright Field) and HAADF (High Angle Annular Dark Field) detectors simultaneously. Low-magnification TEM images (10,000× and 15,000×) revealed that the C-NaOH + H_2_SO_4_ sample is composed of thin sheets of varying sizes (graphene and micrographite nanoplatelets). Using only manual agitation, few edge regions were suitable for analysis, and in these regions, the presence of thin sheets was observed, suggesting that the sample is composed of thin layers (graphene and micrographite nanoplatelets). The use of an ultrasonic bath for 2 min promoted the separation of these layers, resulting in the deposition of several thin sheets (graphene nanoplatelets) of various sizes on the grid. It is important to note that, due to the sheets being rolled, torn, and/or overlapping, the average size obtained represents only an estimate. Size distribution analysis was performed using Gatan Digital Micrograph software, and the particle size distribution histogram was prepared using Origin 8.1. The graphene nanoplates (<5 nm) are nearly transparent and have some visible wrinkles, suggesting that the samples are mainly composed of few-layer graphene (Figure 11). For the graphene nanoplates (>5 nm), they are less transparent due to the higher thickness (Figure 12). The micrographite has a similar grain size of ~1 μm as graphene nanoplates, but its thickness is nearly the same as the bulk graphite.

## 4. Conclusions

The microcrystalline graphite ore sample from Canindé, CE, was subjected to flotation tests combined with chemical purification in a basic medium followed by acid leaching. After this process, the sample exhibited high purity (99% graphite) and strong potential for graphene nanoplate and graphene-like material production, displaying a well-defined crystalline structure and thin particles. This combination of processes disrupted the regular stacking of graphite layers, leading to the formation of graphene nanoplatelets, as evidenced by the change in the Raman spectrum, FTIR, and AFM between the C-Bulk sample and the C-NaOH + H_2_SO_4_-treated sample, AFM and TEM scan measurements showing particle sizes below 20 nm, consistent with graphene and micrographite nanoplatelets. The chemical composition of the C-NaOH + H_2_SO_4_ treatment sample indicates a significant reduction in impurities such as ferrimuscovite due to the removal of elements that constitute this mica. The C-Bulk sample (graphite concentrate) contains 76.6% graphitic carbon, which increases to over 99% after leaching, suggesting highly efficient purification. Acid leaching presents significant advantages, such as lower environmental impact and better control over the morphology and properties of the obtained graphene compared to traditional chemical methods that use HF. Therefore, the data obtained from XRD patterns, Raman spectroscopy, FTIR, AFM, and TEM collectively indicate that the combined purification process (basic + acidic) not only enabled the removal of mineral and metallic impurities but also promoted the reorganization and exfoliation of graphite into thinner layers. This approach allowed us to produce graphene nanoplatelets without the need for sonication or harsh oxidizing agents, representing a promising and low-cost strategy for the preparation of graphene derivatives from natural sources.

## Figures and Tables

**Table 1:** Refinement parameters (Rietveld method) including the lattice parameters, phase, and crystallite size calculated using the Scherrer equation for the C-Bulk and C-NaOH + H_2_SO_4_ treatment samples.

C-Bulk	Chemical Formula	Space Group	Weight Fraction (%)	Lattice Parameters (Å, °)	Cell Volume (Å^3^)	Rwp (%)	GOF (χ^2^)	Crystallite Size (nm)
Muscovite	KAl_2_(AlSi_3_O_10_)(OH)_2_	C 1 2/c 1	0.1	a = 13.20 b = 4.84 c = 14.66 β = 68.89°	875.86	16.9	1.53	19.01
Graphite	C	P 63/m m c	71.4	a = 2.463; c = 6.719	35.330			83.04
Quartz	SiO_2_	P 31 2 1	28.5	a = 4.928; c = 5.519	116.125			42.30
**C-NaOH + H_2_SO_4_ Treatment**	**Chemical Formula**	**Space Group**	**Weight Fraction (%)**	**Lattice Parameters (Å, °)**	**Cell Volume (Å^3^)**	**Rwp (%)**	**GOF (χ^2^)**	**Crystallite Size (nm)**
Graphite	C	P 63/m m c	100.00	a = 2.53777 c = 6.77568	37.791	15.02	1.532	130

**Table 2:** Chemical composition of the samples: graphite ore—Stage 1, C-Bulk—Stage 2, C-NaOH treatment—Stage 3, and C-NaOH + H2SO4 treatment—Stage 4.

Analyses	SiO_2_	Al_2_O_3_	CaO	MgO	K_2_O	TiO_2_	MnO	Fe_2_O_3_	SO_3_	Cl	P_2_O_5_	Co_2_O_3_	Rh_2_O_3_
Method	XRF	XRF	XRF	XRF	XRF	XRF	XRF	XRF	XRF	XRF	XRF	XRF	XRF
Unit	%	%	%	%	%	%	%	%	%	%	%	%	%
Detection Limit	0.01	0.01	0.01	0.01	0.01	0.01	0.01	0.01	0.01	0.01	0.01	0.01	0.01
Stage 1	42.43	18.88	2.19	1.06	7.55	2.69	2.69	23.81	0.34	0.62	0.00	0.00	0.00
Stage 2	31.02	15.65	2.44	0.00	6.32	2.90	0.54	36.16	0.37	0.75	3.67	0.18	0.00
Stage 3	29.75	12.88	2.61	1.06	5.13	3.52	0.34	39.78	0.00	0.00	3.73	0.00	1.22
Stage 4	75.65	3.63	4.70	0.00	3.71	0.00	0.00	0.86	0.00	0.00	11.45	0.00	0.00

## Data Availability

The original contributions presented in this study are included in the article. Further inquiries can be directed to the corresponding author.
